# Supplementary material for: Prognostic Index for Predicting Prostate Cancer Survival in a Randomized Screening Trial: Development and Validation
Source: Cancers (Basel). 2021 Jan 24;13(3):435. doi: 10.3390/cancers13030435 (PMC7865328; doi:10.3390/cancers13030435)
Supplement: Supplementary file 1 [file cancers-13-00435-s001.pdf]

cancers

MDPI

Supplementary materials: Prognostic index for predicting prostate cancer survival in a randomized screening trial: Development and validation

Subas Neupane *, Jaakko Nevalainen, Jani Raitanen MSc, Kirsi Talala, Paula Kujala, Kimmo Taari, Teuvo Tammela, Ewout W. Steyerberg and Anssi Auvinen

Table S1. Prognostic factors for prostate cancer mortality from the prediction model. Estimates from the complementary log-log regression model with their 95% confidence intervals in different time periods.

Characteristics	Regression coefficient, β (95% CI)					
	0-3 years	p-value	3-9 years	p-value	9-20 years	p-value
**Age at diagnosis**	-0.02 (-0.08 to 0.05)	0.581	-0.03(-0.07 to 0.01)	0.195	-0.10 (-0.15 to -0.05)	<0.001
**Study arm**						
Control	Ref		Ref		Ref	
Screening	-0.25 (-6.04 to -5.54)	0.933	1.04 (-2.71 to 4.80)	0.586	-2.08 (-6.43 to 2.28)	0.350
**PSA at diagnosis**	0.15 (-0.18 to 0.48)	0.368	-0.15 (-0.38 to 0.09)	0.221	0.08 (-0.21 to 0.38)	0.582
**EAU Risk group**						
Low	Ref		Ref		Ref	
Intermediate	0.75 (-0.73 to 2.23)	0.322	1.14 (0.59 to 1.69)	<0.001	0.28 (-0.20 to 0.76)	0.259
High	1.94 (0.60 to 3.28)	0.005	1.74 (1.13 to 2.34)	<0.001	0.46 (-0.16 to 1.09)	0.146
**Comorbidity index**						
0	Ref		Ref		Ref	
1+	0.16 (-0.53 to 0.85)	0.646	-0.09 (-0.66 to 0.47)	0.745	-1.08 (-2.23 to 0.06)	0.067
**Primary treatment**						
Radical Prostatectomy	Ref		Ref		Ref	
Radiation	0.09 (-0.75 to 0.93)	0.839	1.51 (1.01 to 2.00)	<0.001	1.33 (0.86 to 1.80)	<0.001
Hormonal	1.56 (0.67 to 2.45)	0.001	2.68 (2.09 to 3.27)	<0.001	2.09 (1.38 to 2.80)	<0.001
Observation	-0.46 (-2.15 to 1.22)	0.591	1.97 (1.26 to 2.68)	<0.001	2.61 (0.89 to 2.33)	<0.001
No treatment	3.19 (1.99 to 4.40)	<0.001	NA		NA	
**Biochemical recurrence**						
No	Ref		Ref		Ref	
Yes	0.77 (0.12 to 1.42)	0.021	2.50 (2.10 to 2.93)	<0.001	2.37 (1.86 to 2.89)	<0.001
Arm x age at diagnosis		0.889		0.598		0.321

NA: Not available.

Table S2. Distribution of prognostic index quintiles for the development period 0-3 years reclassified in new prognostic index quintiles for the validation period 3-6 years.

Prognostic index 0 to 3 years	Prognostic index 3 to 6 years				
	Q1	Q2	Q3	Q4	Q5
**Q1**	445 (31.6%)	850 (60.4%)	86 (6.1%)	25 (1.8%)	1 (0.1%)
**Q2**	589 (42.7%)	155 (11.2%)	270 (19.6%)	302 (21.9%)	64 (4.6%)
**Q3**	196 (13.9%)	298 (21.2%)	716 (50.9%)	52 (3.7%)	146 (10.4%)
**Q4**	39 (2.8%)	80 (5.8%)	212 (15.3%)	479 (34.6%)	576 (41.6%)
**Q5**	23 (1.8%)	14 (1.1%)	114 (8.9%)	517 (40.5%)	608 (47.7%)

Table S3. Risk score at diagnosis for Pca survival based on the complementary log-log regression model among Pca diagnosis cases among both arm of the trial.

Characteristics	Categories	β†	Risk score§
**Age at diagnosis (Years)**			
	≤60	1.38	45
	61-70	1.22	40
	71-75	1.05	35
	≥76	ref	0
**PSA at diagnosis (ng/ml)**			
	≤19.9	ref	0
	≥20.0	0.14	5
**EAU Risk group**			
	Low	ref	0
	Intermediate	0.76	25
	High	1.62	50

† Regression coefficients from complementary log-log model. § Risk score at baseline calculated dividing each beta coefficients by the sum of the highest beta coefficient of each variables and then multiplied by 100. (Scores are rounded).

Table S4. Risk score at diagnosis for Pca survival based on the complementary log-log regression model among Pca diagnosis cases in the control arm (complete case analysis).

Characteristics	Categories	β†	Risk score§
**Age at diagnosis (Years)**			
	≤60	1.07	40
	61-70	0.94	35
	71-75	0.67	25
	≥76	ref	0
**PSA at diagnosis (ng/ml)**			
	≤19.9	ref	0
	≥20.0	0.27	10
**EAU Risk group**			
	Low	ref	0
	Intermediate	0.66	25
	High	1.36	50

† Regression coefficients from complementary log-log model. § Risk score at baseline calculated dividing each beta coefficients by the sum of the highest beta coefficient of each variables and then multiplied by 100. (Scores are rounded).

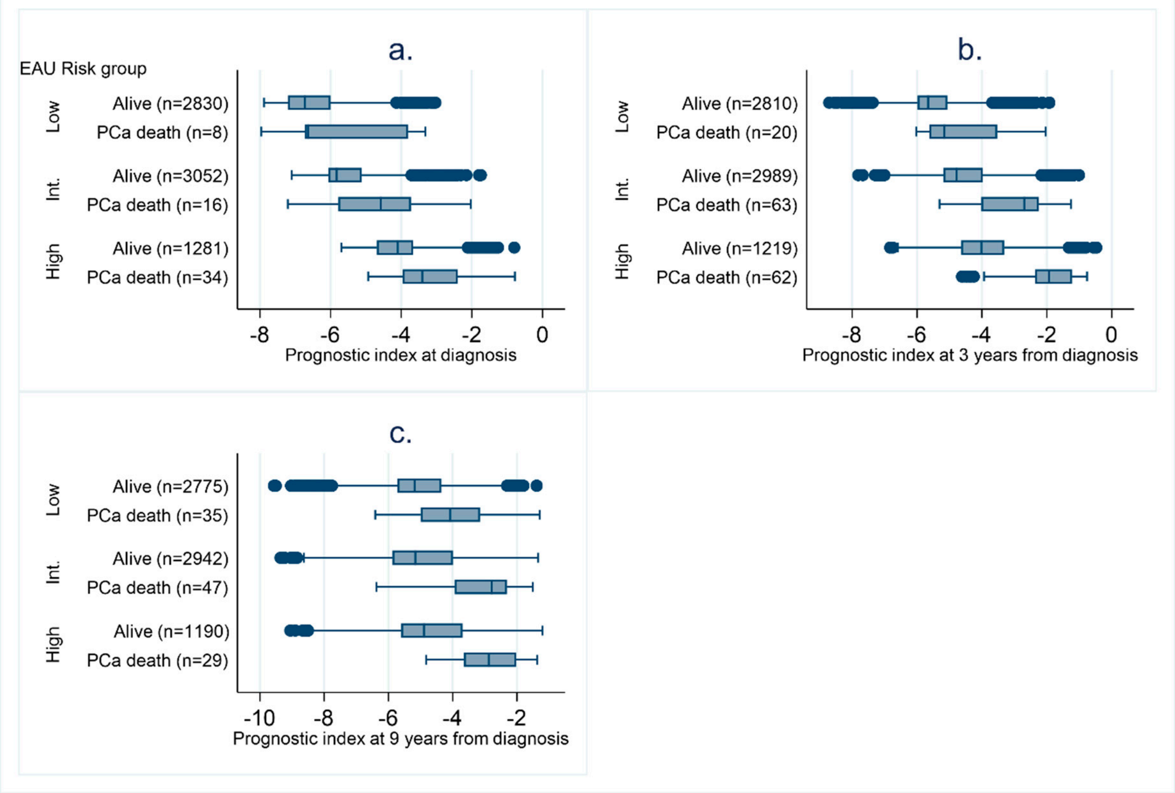

Figure S1. Box plots showing the distribution of the prognostic index by EAU risk groups and by period (a) 0 to 3 years, (b) 3 to 9 years and (c) 9 to 20 years.

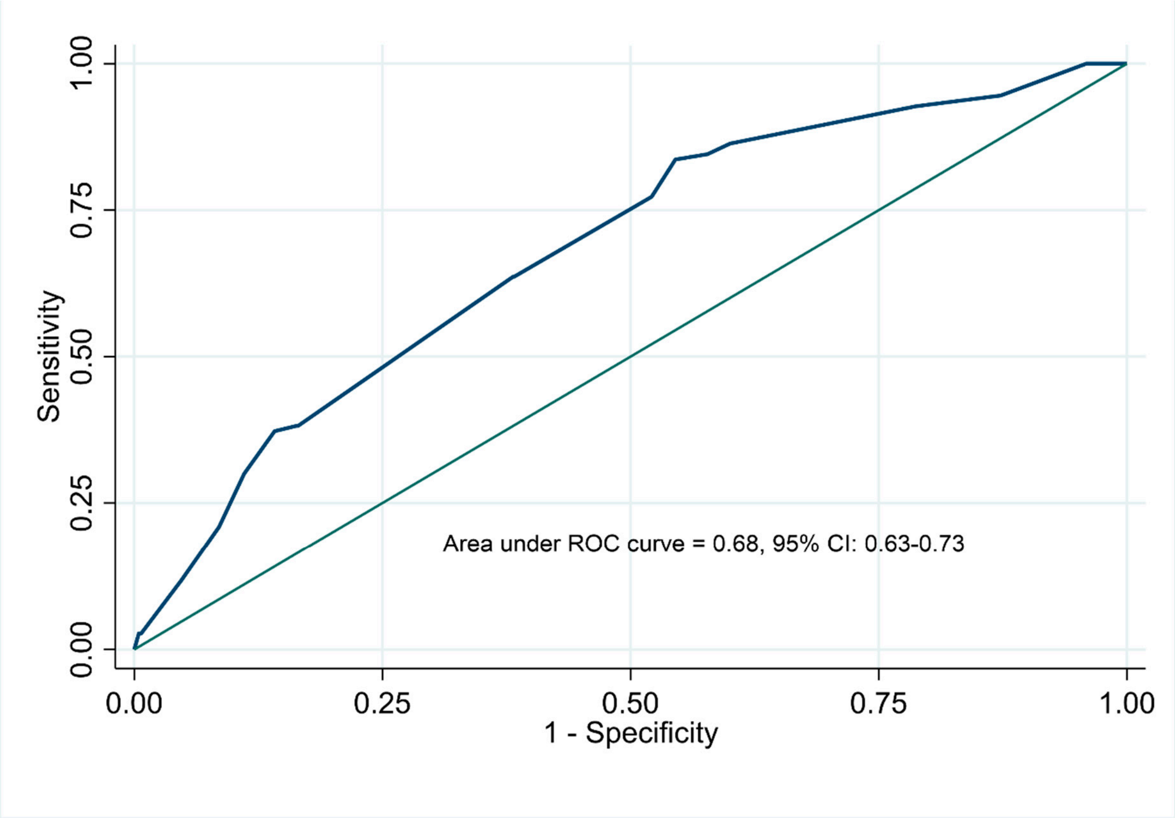

Figure S2. ROC curve for the simplified risk score at diagnosis.

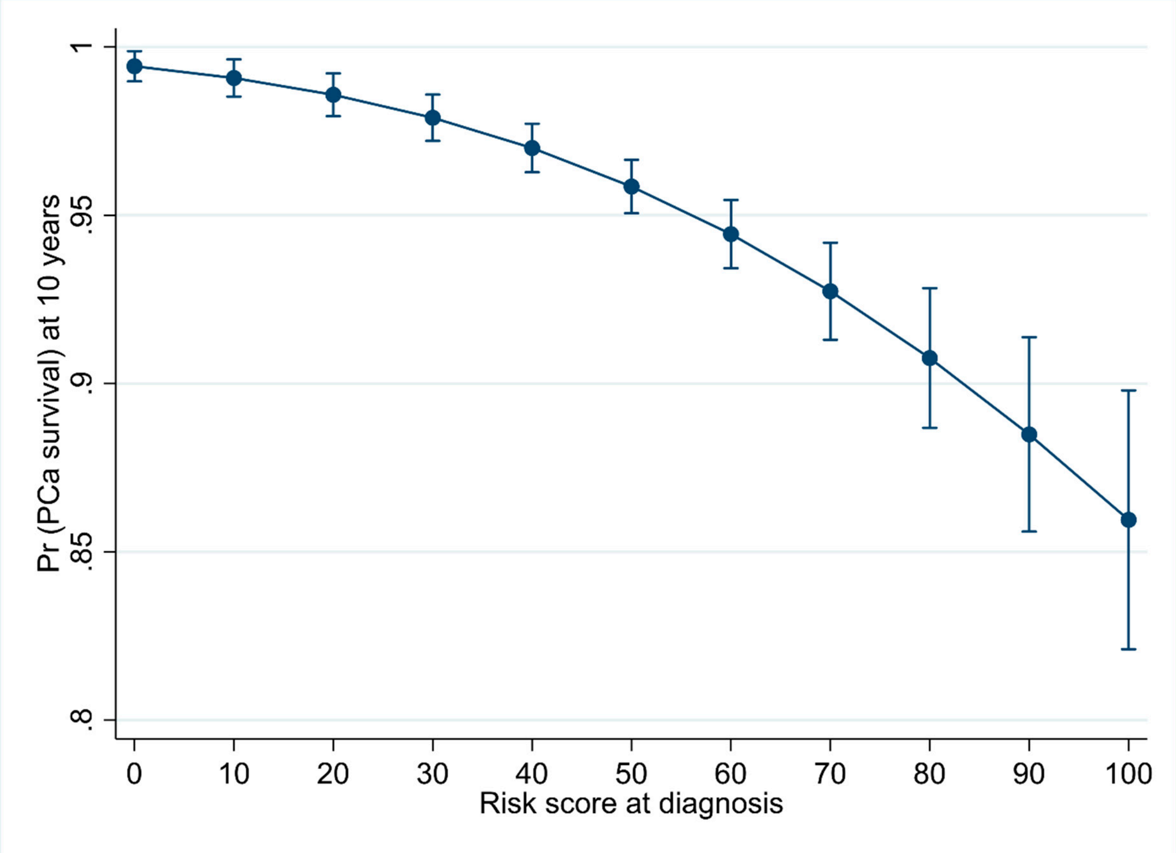

Figure S3. Predicted probability of PCa survival at 10-year from diagnosis among controls in a Finnish randomized screening trial (complete case analysis).
